# Applicability of the Padua scale for Chinese rheumatic in-patients with venous thromboembolism

**DOI:** 10.1371/journal.pone.0278157

**Published:** 2022-12-16

**Authors:** Qing Peng, Xixi Chen, Yaxin Han, Guo Tang, Jiajun Liu, Yan Liu, Qiao Zhou, Li Long

**Affiliations:** 1 Department of Rheumatology, Chengdu Second People’s Hospital, Chengdu, China; 2 Department of Rheumatology, Sichuan Provincial People’s Hospital, University of Electronic Science and Technology of China, Chengdu, China; 3 Chinese Academy of Sciences Sichuan Translational Medicine Research Hospital, Chengdu, China; 4 The People’s Hospital of Wenjiang, Chengdu, China; 5 Department of Nephrology, The People’s Hospital of Bishan District, Chongqing City, China; 6 Zunyi Medical University, Zunyi, Guizhou, China; Ataturk University Faculty of Medicine, TURKEY

## Abstract

**Objective:**

To investigate the risk factors for venous thromboembolism (VTE) in hospitalized patients with rheumatic diseases in China. The efficacy of the Padua scale was evaluated and an improved model for predicting VTE in hospitalized patients with rheumatic diseases was developed.

**Methods:**

Records of 2282 patients hospitalized in the department of rheumatology of the Sichuan Provincial People’s Hospital were retrospectively reviewed. The risk factors for VTE were analyzed. The efficacy of the Padua scale was evaluated, Padua-combined prediction model and the independent risk factor-combined prediction model for predicting VTE were assessed using the receiver operating curve (ROC).

**Results:**

A total of 50 patients in the VTE group and 2232 in the non-VTE group were included. Antiphospholipid syndrome (APS), VTE history, a hospital stay of over 3 days, high D-dimer (D-D), and decreased serum albumin were independent risk factors for VTE. APS was very closely associated with VTE (OR = 19.446). Padua scores in the VTE group and the non-VTE group were 3 (2, 6) and 2 (1, 2) points, respectively (*p* < 0.05), and the proportion of high-risk patients were 48.0% and 7.4%, respectively (*p* < 0.05). The incidence of VTE in the high-risk (Padua score ≥4) and low-risk (Padua score <4) groups was 12.7% and 1.2%, respectively (*p* < 0.05). The area under curve (AUC) of the Padua scale, Padua combined prediction model (Padua scale along with D-D and serum albumin), and the independent risk factor-combined prediction model was 0.771, 0.836, and 0.873, respectively.

**Conclusion:**

The Padua scale has limited predictive efficacy of VTE in hospitalized rheumatic patients. The independent risk factor-combination prediction model was superior in predicting VTE compared to Padua scale and Padua-combined prediction model.

## 1 Introduction

Venous thromboembolism (VTE) is a thrombotic disease of the venous system, which primarily includes the deep venous thrombosis (DVT) of the lower extremities and pulmonary thromboembolism (PTE). A 10-year study involving 530 hospitals in China showed a dramatic increase in the prevalence of VTE, from 3.2/100000 people in 2007 to 17.5/100000 people in 2016, which is lower than that in the United States (99/100000), Canada (138/100000), and South Korea (29.2/100000) during the same period [1–3]. However, the in-hospital mortality rate for VTE in China was comparable to that in other developed countries [4].

Studies have shown that the incidence of VTE is high among patients with rheumatic diseases. For example, the incidence of VTE is 7.97% in patients with anti-neutrophil cytoplasmic antibodies (ANCA) -associated vasculitis, 7.29% in patients with systemic lupus erythematosus (SLE), 4.03% in patients with idiopathic inflammatory myositis (IIM), and 2.18% in patients with rheumatoid arthritis (RA) and primary Sjogren’s syndrome (pSS) [5–10]. Serum albumin, D-dimer (D-D) levels, and platelets (PLT) are closely related to the occurrence of VTE [11, 12]. However, there were few studies on the occurrence of VTE in patients with rheumatic diseases. Glucocorticoid therapy is a key agent for inducing remission in the acute stage of rheumatic diseases. Previous large-scale studies have indicated that the use of glucocorticoids could increase the risk of VTE [13, 14].

In 2010, Barbar et al. proposed a VTE risk assessment model, also known as the Padua predictive score scale [15]. In 2012, the American College of Chest Physicians (ACCP) guidelines recommended the Padua scale for VTE risk assessment in non-surgical inpatients [16]. In 2018, the Padua scale was recommended in the Chinese Guidelines for the Prevention and Treatment of Thrombotic Diseases for assessing the risk of VTE in inpatients in internal medicine [17]. At present, there are insufficient studies on the application of the Padua scale in patients hospitalized with rheumatic diseases at home and abroad. Thus, this study aimed to evaluate the validity of the Padua scale for predicting VTE in hospitalized patients with rheumatic diseases and to find out an improved model with high predictive efficacy.

## 2 Materials and methods

### 2.1 Study subjects

This study is a retrospective cross-sectional study. Patients who were admitted to the Department of Rheumatology and Immunology of Sichuan Provincial People’s Hospital between September 5, 2018 and December 27, 2020 and met the inclusion criteria were selected. The study was approved by the Ethics Committee of the Sichuan Provincial People’s Hospital (No. 2020–362) and all participants provided consent. During the data collection process, we could obtain information to identify individual participants.

### 2.2 Diagnostic methods for VTE

DVT is diagnosed by deep vein ultrasound and/or deep vein angiography of the lower extremity, and PTE is diagnosed by computed tomography pulmonary arteriography (CTPA). All Patients with VTE included in this study were diagnosed with symptomatic VTE after a comprehensive screening and then confirmed after clinically suspected VTE.

### 2.3 Inclusion criteria and exclusion criteria

Inclusion criteria were as follows: (1) age ≥18 years; (2) hospitalized for rheumatic diseases; (3) a hospital stay of over 3 days. Exclusion criteria were as follows: (1) individuals who have received anticoagulant therapy at admission; (2) patients who were unable to confirm a new onset of VTE during hospitalization; (3) patients who didn’t meet the diagnostic criteria for rheumatism; (4) patients without complete clinical data for evaluation.

### 2.4 Classification criteria and disease activity criteria for rheumatic diseases

All the classification criteria for rheumatic diseases are based on current internationally recognized classification criteria. The evaluation methods of disease activity were systemic lupus erythematosus disease activity index 2000 (SLEDAI-2000) for SLE, 28-joint disease activity score (DAS28) for RA, Birmingham vasculitis activity score 2003 (BVAS-2003) for systemic vasculitis, visual analogue scale (VAS) for IIM, EULAR Sjögren’s syndrome disease activity index (ESSDAI) for pSS, and system score for adult-onset Still’s disease (AOSD) [17].

### 2.5 Data acquisition

11 risk factors for the Padua scale, serum albumin, D-dimer, and platelet (PLT) levels were collected within a week before discharge or the occurrence of VTE. Each patient was scored for thrombotic risk by a professionally trained rheumatologist, in strict accordance with the criteria of the Pauda Scale.

### 2.6 Case grouping

Patients were divided into the VTE and non-VTE groups according to whether VTE was diagnosed during hospitalization. Patients with the Padua score ≥4 were enrolled in the high-risk group, while others were enrolled in the low-risk group.

### 2.7 Statistical analysis

Excel software was used to record patients’ information and establish the database. Statistical analysis was performed using SPSS Statistics 26.0 and MedCalc statistical software. Measurement data with normal distribution were expressed as $\bar{x}\pm s$ and the t-test was used for comparison between the two groups. Measurement data with non-normally distribution were expressed as *Median* (*P*_25_, *P*_75_), and the nonparametric Mann-Whitney U rank-sum test was used for comparison between the two groups. Categorical data were expressed as frequencies (percentages), and a comparison between the two groups was performed using the *χ*^2^ tests or Fisher’s exact test. Logistic regression was used to analyze independent risk factors for the occurrence of VTE, and odds ratios (ORs) and 95% confidence intervals (CIs) were calculated. The area under the receiver operating characteristic (ROC) curve (AUC) was used to estimate the sensitivity and specificity of the Padua scale and an improved model for the occurrence of VTE in patients with the Padua scale. A model with AUC values above 0.7 is considered to be accurate. The differences between the AUC of the three models were compared using the Delong method. *p*< 0.05 was considered statistically significant.

## 3 Results

### 3.1 Distribution in VTE and non-VTE groups

From September 5, 2018 to December 27, 2020, a total of 5128 inpatients were enrolled. Among them, 1,840 patients had a hospital stay of less than 3 days, 437 patients had received anticoagulant therapy at admission, 301 patients did not meet the diagnostic criteria for rheumatic diseases, 146 patients had insufficient personal information and data, and 122 patients were aged less than 18 years. Finally, 2282 patients who met the inclusion criteria were selected for further analysis, which included 50 (2.2%) cases in the VTE group and 2232 (97.8%) cases in the non-VTE group.

In the VTE group, there were 38 (76%) cases of DVT, 8 (16%) cases of PTE, and 4 (8%) cases of DVT combined with PTE. The disease distribution in the two groups is shown in Table 1. SLE accounted for the highest percentage (34.0%) in the VTE group, and 4 of 17 cases had combined with APS. In the non-VTE group, RA accounted for the highest percentage of 24.2, followed by SLE (18.2%). Among SLE patients, 6 cases had both SE and APS.

**Table 1:** The disease distribution in the VTE and non-VTE groups (n = 2282).

Disease type	VTE group	Non-VTE group	Total
	(n = 50)	(n = 2232)	(n = 2282)
RA, n (%)	9 (18.0)	541 (24.2)	550 (24.1)
SLE, n (%)	17 (34.0)	406 (18.2)	423 (18.5)
pSS, n (%)	2 (4.0)	292 (13.1)	294 (12.9)
OS, n (%)	2 (4.0)	173 (7.8)	175 (7.7)
IIM, n (%)	5 (10.0)	156 (7.0)	161 (7.1)
Gout, n (%)	3 (6.0)	134 (6.0)	137 (6.0)
SpA, n (%)	2 (4.0)	131 (5.9)	133 (5.8)
UCTD, n (%)	1 (2.0)	106 (4.7)	107 (4.7)
MCTD, n (%)	0 (0)	15 (0.7)	15 (0.7)
Vasculitis, n (%)	8 (16.0)	109 (4.9)	117 (5.1)
OA, n (%)	0 (0)	96 (4.3)	96 (4.2)
SSc, n (%)	0 (0)	51 (2.3)	51 (2.2)
AOSD, n (%)	1 (2.0)	22 (1.0)	23 (1.0)

RA, rheumatoid arthritis, SLE, systemic lupus erythematosus, pSS, primary Sjogren’s syndrome, OS, overlap syndrome, IIM, Idiopathic inflammatory myositis, SpA, Spondyloarthritis, UCTD, undifferentiated connective tissue, MCTD, mixed connective tissue disease, OA, osteoarthritis, SSc, systemic sclerosis, AOSD, adult-onset Still’s disease. Categorical data were expressed as frequencies (percentages).

Disease activity scores for rheumatic diseases in the VTE group are shown in Table 2. 2 of 3 patients had acute gouty arthritis attacks. and 2 patients with undifferentiated spondyloarthritis (SpA) were clinically evaluated in the active stage of the disease. All patients with rheumatic diseases in the VTE group were in the active stage of the disease.

**Table 2:** Evaluation of disease activity for rheumatic diseases in the VTE group (n = 50).

Assessment method	Average score [*M* (*P*_25_, *P*_75_)]	Disease activity
SLEDAI	16.0 (6.0,22.0)	highly active
DAS28	4.9 (4.3,6.6)	moderate active
BVAS	16.0 (13.3,18.3)	active
VAS	6.0 (5.5,8.5)	moderate active
ESSDAI	4.0 (2.0,8.0)	highly active
AOSD score	3.0 (1.3,4.8)	active

SLEDAI, systemic lupus erythematosus disease activity index, DAS28, 28-joint disease activity score, BVAS, Birmingham vasculitis activity score, VAS, visual analogue scale, ESSDAI, EULAR Sjögren’s syndrome disease activity index, AOSD, adult-onset Still’s disease.

### 3.2 Analysis of VTE-related factors in rheumatic inpatients

#### 3.2.1 Analysis of high-risk factors for VTE in hospitalized rheumatic patients

Among the 2282 hospitalized patients included in this study, no antithrombin deficiency, protein C or protein S deficiency, and mutation of V Leiden factor and prothrombin G20210A were found by review of medical history and laboratory tests. All known cases of thromboembolism were patients with APS, with 10(0.4%) cases in total. A hospital stay of over 3 days, APS, previous VTE history, respiratory or heart failure, recent trauma or surgery, glucocorticoid therapy, age, serum albumin and D-D levels were significant differences between the VTE group and the non-VTE group (*p* < 0.05), as shown in Table 3.

**Table 3:** Univariate analysis for VTE among the hospitalized rheumatic patients (n = 2282).

Category, n (%)		VTE group (n = 50)	Non-VTE group (n = 2232)	Total (n = 2282)	*χ*^2^/*Ζ* value	*p* value
Sex, n(%)	Male	17 (34.0)	539 (24.1)	556 (24.4)	2.575	0.109
	Female	33 (66.0)	1693 (75.9)	1726 (75.6)		
Active malignancy, n(%)	yes	1 (2.0)	18 (0.8)	19 (0.8)	−	0.345**
	no	49 (98.0)	2214 (99.2)	2263 (99.2)		
Hospital stay > 3 days, n(%)	yes	12 (24.0)	40 (1.8)	52 (2.3)	102.687	<0.001
	no	38 (76.0)	2192 (98.2)	2230 (97.7)		
Thrombophilia, n(%)	yes	4 (8.0)	6 (0.3)	10 (0.4)	−	<0.001**
	no	46 (92.0)	2226 (99.7)	2272 (99.6)		
Previous VTE history, n(%)	yes	7 (14.0)	27 (1.2)	34 (1.5)	−	<0.001**
	no	43 (86.0)	2205 (98.8)	2248 (98.5)		
Respiratory/heart failure, n(%)	yes	7 (14.0)	89 (4.0)	96 (4.2)	9.808	0.002
	no	43 (86.0)	2143 (96.0)	2186 (95.8)		
Acute myocardial/cerebral infarction, n(%)	yes	3 (6.0)	79 (3.5)	82 (3.6)	0.292	0.589
	no	47 (94.0)	2153 (96.5)	2200 (96.4)		
Recent trauma/surgery, n(%)	yes	6 (12.0)	55 (2.5)	61 (2.7)	13.624	<0.001
	no	44 (88.0)	2177 (97.5)	2221 (97.3)		
Acute infection/rheumatism, n(%)	yes	50 (100)	2232 (100)	2282 (100.0)	−	−
	no	0 (0)	0 (0)	0 (0)		
Glucocorticoid treatment, n(%)	yes	42 (84.0)	1406 (63.0)	1448 (63.5)	9.306	0.001
	no	8 (16.0)	826 (37.0)	834 (36.5)		
Age, (years)	≥70	11 (22.0)	291 (13.0)	302 (13.2)	3.421	0.064
	<70	39 (78.0)	1941 (87.0)	1980 (86.8)		
BMI, (kg/m^2^)	≥30	3 (6.0)	65 (2.9)	68 (3.0)	0.722	0.396
	<30	47 (94.0)	2167 (97.1)	2214 (97.0)		
Age, (year)	-	61.0 (38.8–69.0)	52.5 (41.0–64.0)	-	-2.203*	0.028
PLT (×10^9^/L)	-	216.0 (113.5, 297.0)	203.0 (145.0, 273.0)	-	−0.225*	0.822
D-D (mg/L)		3.9 (0.9, 8.6)	0.8 (0.3, 2.0)	-	−5.745*	<0.001
Serum albumin (g/L)		30.0 (25.4, 34.0)	35.5 (31.1, 39.0)	-	−5.573*	<0.001

VTE, venous thromboembolism, APS, anti-phospholipid antibody syndrome, BMI, body mass index, PLT, platelet, D-D, D-dimer.

* represents the *Z-*value of the non-parametric test.

**p is the *p*-value obtained by Fisher’s exact test.

Multivariate logistic regression analysis was performed with the occurrence of VTE event during hospitalization as the dependent variable, and a hospital stay > 3 days, APS, previous VTE history, glucocorticoid treatment, D-D and serum albumin levels as the independent variables. Results of the regression analysis showed that a hospital stay > 3 days, previous history of VTE, APS, high D-D levels, and low serum albumin levels were independent risk factors for VTE in hospitalized rheumatic patients, as shown in Table 4. APS was the most closely related risk factor for the occurrence of VTE (OR = 19.446). Further analysis revealed an increased risk of VTE in hospitalized patients with lower serum albumin levels. The risk of VTE in patients with serum albumin ≤20g/L was 13.5-fold higher than that in patients with the normal level of serum albumin (≥40g/L), as shown in Table 5.

**Table 4:** Multivariate binary logistic regression analysis for VTE among the hospitalized rheumatic patients.

Risk factors	B	SE	Wals	df	*p*-value	OR	95% CI
D-D	0.033	0.014	5.226	1	0.022	1.033	1.005–1.063
Serum albumin	-0.098	0.025	15.359	1	0.000	0.907	0.864–0.952
Hospital stay > 3 days	2.555	0.401	40.687	1	0.000	12.877	5.872–28.238
Previous VTE history	2.661	0.503	28.043	1	0.000	14.312	5.345–38.322
APS	2.968	0.784	14.314	1	0.000	19.446	4.180–90.467

D-D, D-dimer, VTE, venous thromboembolism, APS, anti-phospholipid antibody syndrome.

**Table 5:** Correlational analysis between serum albumin level and the risk for the occurrence of VTE.

Serum albumin level (g/L)	B	SE	Wals	df	*p*-value	OR	95% CI
≥40	−	−	31.234	5	0.000	1.000	−
35−39.99	0.295	0.710	0.172	1	0.678	1.343	0.33−5.394
30−34.99	1.440	0.632	5.192	1	0.023	4.221	1.223−14.571
25−29.99	2.220	0.637	12.140	1	0.000	9.205	2.641−32.083
21−24.99	2.469	0.699	12.476	1	0.000	11.812	3.001−46.491
≤20	2.603	0.835	9.726	1	0.002	13.500	2.630−69.298

#### 3.2.2 Padua scale scoring and risk analysis for VTE occurrence

The Padua score of 2282 inpatients was 2.08 ± 1.11, with a maximum score of 10 and a minimum score of 1. The Padua scores were higher in the VTE group than those in the non-VTE group [3 (2, 6) vs. 2 (1, 2), *Ζ* = 6.961, *p* < 0.001].

**Fig 1:**
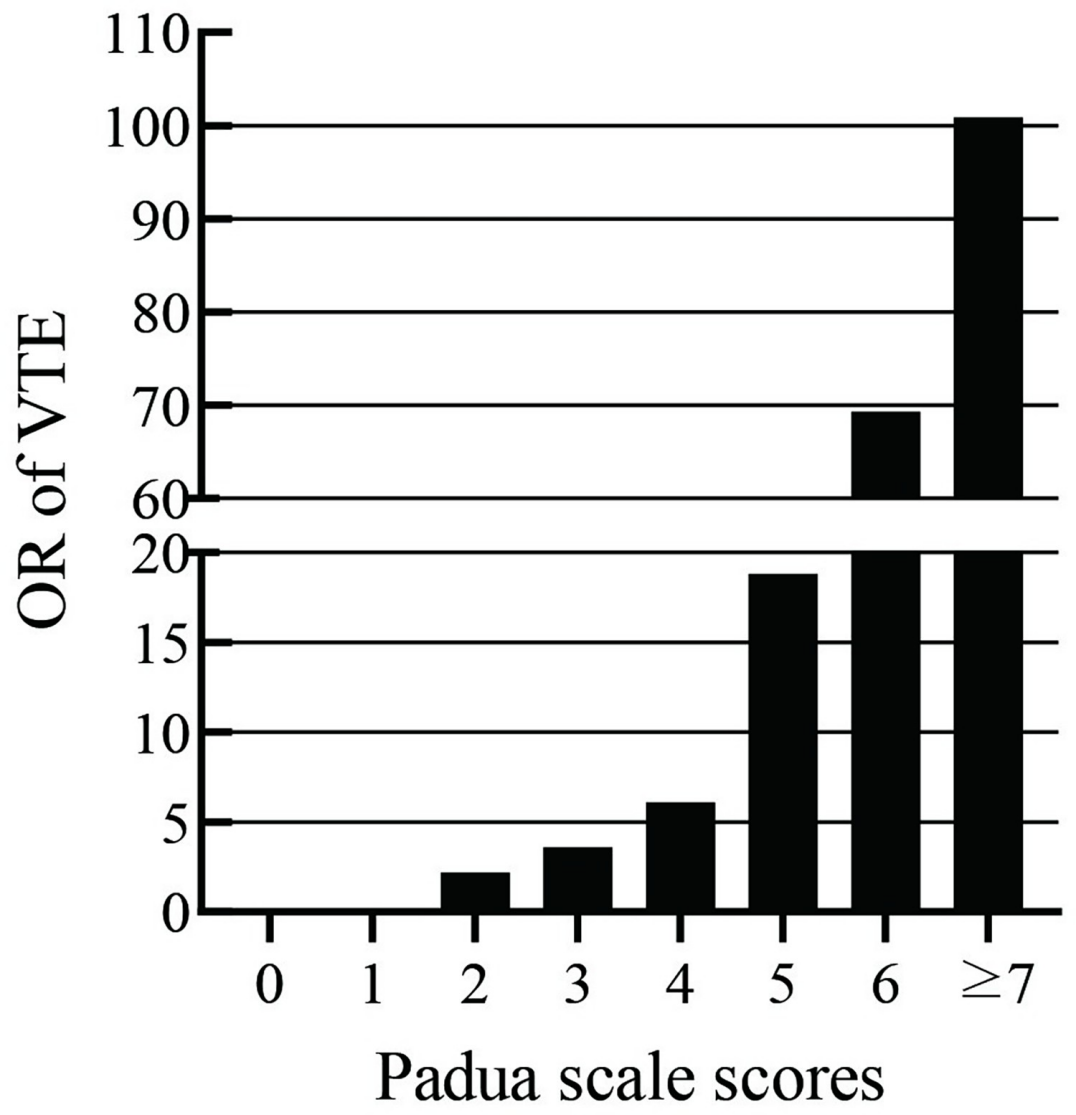
Padua scale scores and risk of VTE. VTE, venous thromboembolism, OR, odds ratio.

Among the 2282 hospitalized patients, 189 (8.3%) patients were at high risk for VTE. The proportion of patients with VTE was 12.7% in the high-risk group and 1.2% in the low-risk group. The higher the Padua scale level, the higher the percentage of VTE identification, which was statistically significantly different (*χ*^2^ = 100.882, *p* < 0.001). Additionally, with the increase in the Padua score, the possibility of VTE occurrence was also higher (shown in Fig 1).

#### 3.2.3 Analysis of the clinical value of the Padua scale for predicting VTE

**Fig 2:**
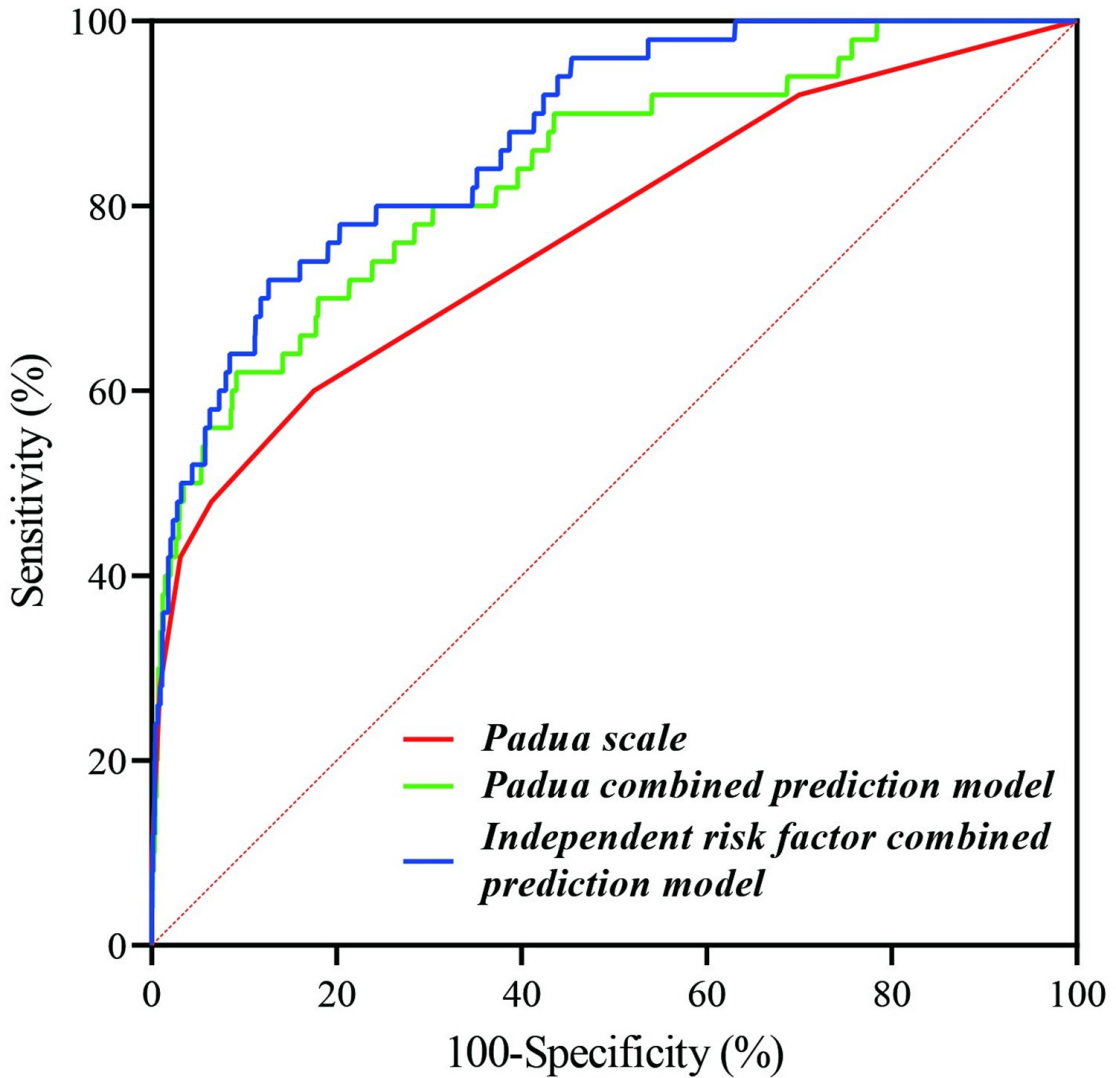
ROC curves of the Padua scale, Padua combined-prediction model, and independent risk factor-combined prediction model. Padua combined prediction model refers to the Padua scale combined with D-D and serum albumin. The independent risk factor-combined prediction model refers to the combination of five risk factors as follows: a hospital stay > 3 days, previous VTE history, APS, high D-D, and low serum albumin levels.

To avoid the effect of prophylactic measures on the outcome of VTE, 104 patients who underwent preventive anticoagulant therapy at hospitalization were excluded from all the ROC curve analyses. The efficacy of the Padua scale for VTE prediction is shown in Table 6 and Fig 2.

**Table 6:** Efficacy of the Padua scale, Padua-combined prediction model, and independent risk factor-combined prediction model for predicting risk of VTE.

Model	AUC	95% CI	Youden index	Sensitivity	Specificity	Threshold value
Padua scale	0.771	0.753−0.789	0.415	48.0%	93.5%	3
Padua-combined prediction model	0.836	0.820−0.851	0.528	62.0%	90.8%	-
Independent risk factor-combined prediction model	0.873	0.859–0.887	0.594	72.0%	87.4%	-

In this study, D-D and low serum albumin levels were found to be independent risk factors for VTE in hospitalized rheumatic patients. The Padua scale combined with D-D and serum albumin levels was defined as the Padua-combined prediction model, shown in Table 6 and Fig 2. The result showed that the Padua-combined prediction model had a better predictive value than the Padua scale alone (*Ζ* = 4.202, *p* < 0.001).

Based on the thrombus assessment model and the multivariate analysis, five risk factors of a hospital stay > 3 days, previous VTE history, APS, high D-D and low serum albumin levels were combined with the Padua scale to develop a new prediction model which was defined as the independent risk factor-combined prediction model. The clinical value of this model for predicting the occurrence of VTE in rheumatic patients was evaluated, as shown in Table 6. The efficacy of the independent risk factor-combined prediction model for predicting the risk of VTE was better than that of the Padua scale (*Ζ* = 3.774, p < 0.001) and the Padua-combined prediction model (*Ζ* = 1.999, *p* = 0.045), as shown in Fig 2.

## 4 Discussion

VTE is characterized by high morbidity, high underdiagnosis, and high mortality. It is a serious complication during hospitalization and has become a global public health problem. At present, the incidence of VTE in inpatients with rheumatic diseases has not been reported both at home and abroad. This study was the first to investigate that the overall incidence of VTE in inpatients with rheumatic diseases was at 2.2%. For economic reasons, only symptomatic VTE was comprehensively screened in this study, which may miss some with asymptomatic VTE. Thus, prospective studies could be conducted in the future to investigate the incidence of asymptomatic VTE.

In this study, APS as a non-inflammatory autoimmune disease was the only observed thrombophilic disorder. DVT is the most common manifestation of APS, accounting for 31.7% of the total cases [18]. In this study, all 10 APS patients combined with SLE. 4 (40%) cases combined with VTE received anticoagulant therapy. And 4 (40%) cases without VTE also received anticoagulant therapy. And other 2 cases didn’t receive prophylactic anticoagulant therapy because of anticoagulant contraindication. Our study showed that APS is the independent risk factor most closely associated with VTE. Thus, patients with APS should be given more attention and prophylactic anticoagulant therapy may improve the outcome of VTE.

Because all patients in the VTE group were in the active stage of rheumatic disease, patients treated with glucocorticoids in the VTE group (84.0%) were higher than those in the non-VTE group (61.7%). Unfortunately, in our study, the disease activity assessment was not performed in the non-VTE group. Patients with active rheumatism are in a high inflammation state which may promote thrombosis by affecting several key factors of coagulation, including upregulation of various procoagulant substances to activate coagulation, reduction of anticoagulant factors to inhibit fibrinolytic system, inhibition of fibrin clearance, and activation of platelets [19–21]. Glucocorticoid use was not an independent risk factor for VTE in hospitalized rheumatic patients, which may be related to the fact that glucocorticoids can control the high inflammatory state of rheumatism and reduce the occurrence of thrombosis.

A prospective cohort study that included 15792 adults found that low serum albumin was a moderate marker of increased risk of VTE [12], but no relationship has been found between serum albumin and VTE in patients hospitalized with rheumatic diseases. In our study, low serum albumin level was found to be independently associated with the occurrence of VTE. The risk of VTE increased progressively with a decrease in serum albumin, with a 9.5% (95% CI: 4.8%-13.6%) increase in the risk of VTE for every 1 g/L decrease in serum albumin. These results were consistent with the findings of Folsom et al. [12].

This retrospective study validated the efficacy of the Padua scale for venous thrombosis in hospitalized patients with rheumatic diseases. The results showed that the risk of VTE is significantly higher in rheumatic inpatients with high-risk Padua scores than in those with low-risk Padua scores, and the risk of VTE increased with an increase in Padua scores. Additionally, the incidence of VTE was higher in patients in the high-risk group than in those in the low-risk group. The VTE patients in the high-risk group were 14.8%, higher than the 11.0% reported in the study by Barbar et al. [15] and 3.5% in a 2014 study [22], which indicated a high incidence of VTE in high-risk patients with rheumatic diseases and also revealed the Padua scale could predict the occurrence of VTE in these patients.

However, in this study, rheumatic patients with a high-risk VTE determined by the Padua scale was only 8.3%, significantly lower than patients with a high risk of cancer (46.3%), acute stroke (40.3%), respiratory diseases (43.3%), and congestive heart failure (54.9%) [23]. The AUC for the Padua scale in our study was 0.771, which was close to the value reported by Zhou et al. [24]. The above results indicated that the Padua scale had a limited value for VTE in hospitalized patients with rheumatic diseases. Possible reasons were as follows: (1) The proportion of risk factors in the Padua scale for hospitalized rheumatic patients was generally low. Except for acute infection and/or rheumatic diseases, glucocorticoid therapy and age ≥70 years, the other 8 risk factors accounted for only 0.4%-4.2%, which was significantly lower than that in internal medicine inpatients reported by Zhou et al. [24]. In addition, once a patient occurs a malignancy, an acute heart attack or an ischemic stroke during hospitalization, she/he would be shifted to other departments. Patients who took anticoagulants a month before hospitalization were excluded from this study. (2) Our study found that the optimal threshold for the Padua scale was 3, which is consistent with the results of several studies [25]. The sensitivity was 48%, indicating that even at an optimal threshold, the Padua scale still classified more patients at high risk of VTE as low risk. It was further verified that the Padua scale had poor predictive efficacy for rheumatic patients. (3) This study is a single-center retrospective cross-sectional study, which inevitably leads to bias in assessing the risk of VTE. In addition, The cases we included were symptomatic VTE, so there is a possibility of missing asymptomatic VTE. In the future, multi-center and prospective studies should be performed, and the predictive efficacy of the Padua scale should be improved for VTE in hospitalized rheumatic patients.

Owing to the poor predictive efficacy of the Padua scale and the findings of the previous study [26] suggest that the VTE assessment model for internal medicine inpatients should include baseline serum levels. Therefore, we included serum albumin and D-D based on the Padua scale and analyze the prediction efficacy of the Padua-combined prediction model. Our findings revealed that the Padua-combined prediction model had a higher AUC than the Padua scale (*p* < 0.001) and sensitivity was increased from 48% to 62%, while there was no significant decrease in specificity. These results showed suggested that the Padua-combined prediction model had better predictive efficacy than Padua scores for VTE in rheumatic inpatients.

Although the diagnostic value of the Padua-combined prediction model was slightly higher than that of the Padua scale, the sensitivity of the Padua-combined prediction model was still poor. Therefore, we created a new prediction model consisting of independent risk factors and analyzed its prediction efficiency. The results showed that the predictive value of the independent risk factor-combined model was superior to both the Padua-combined prediction model and the Padua scale. This was the first attempt to improve a new VTE assessment model based on the Padua scale integrating the characteristics of rheumatic patients, including serum albumin, D-D levels, and other serological indicators. The sensitivity and specificity of this model were both over 70%, and the specificity was higher than sensitivity, which indicated that this model could better distinguish between patients at high risk of VTE, and compensate for the weaknesses of the Padua scale. However, we only provide a preliminary framework for this model at present, and further studies are needed to validate the specific value of each risk factor and its efficiency in the definition of high- and low-risk patients in the model. Prospective studies in multi-center hospitalized patients with rheumatic diseases should be performed for further verification. Until validation of this model, it is recommended that the Padua scale combined with D-D and serum albumin levels be used to assess the risk of VTE in hospitalized rheumatology patients.

## 5 Conclusion

The Padua scale has limited predictive efficacy of VTE in hospitalized rheumatic patients. The independent risk factor-combination prediction model was superior in predicting VTE compared to Padua scale and Padua-combined prediction model.
